# Regulation of Interaction Between the Upper and Lower Airways in United Airway Disease

**DOI:** 10.3390/medsci7020027

**Published:** 2019-02-11

**Authors:** Akira Kanda, Yoshiki Kobayashi, Mikiya Asako, Koichi Tomoda, Hideyuki Kawauchi, Hiroshi Iwai

**Affiliations:** 1 Department of Otolaryngology, Head and Neck Surgery, Kansai Medical University, Hirakata 573-1010, Japan; kobayosh@hirakata.kmu.ac.jp (Y.K.); asako@hirakata.kmu.ac.jp (M.A.); tomodak@hirakata.kmu.ac.jp (K.T.); iwai@takii.kmu.ac.jp (H.I.); 2 Allergy Center, Kansai Medical University, Hirakata 573-1010, Japan; 3 Department of Otorhinolaryngology, Shimane University Faculty of Medicine, Izumo 693-0021, Japan; tomodak@hirakata.kmu.ac.jp

**Keywords:** allergic rhinitis, asthma, eosinophil, nasal-bronchial reflex, united airway disease

## Abstract

The concept of united airway disease comprises allergic rhinitis (AR) with asthma, and eosinophilic chronic rhinosinusitis (ECRS) with asthma. It embodies a comprehensive approach to the treatment of upper and lower airway inflammation. The treatment of upper airway inflammation reduces asthma symptoms and decreases the dose of inhaled corticosteroids (ICS) necessary to treat asthma. However, little is known about the mechanisms of interaction between upper and lower airway inflammation. Here we review these mechanisms, focusing on neural modulation and introduce a novel therapeutic approach to united airway disease using a fine-particle ICS. Our understanding of the relationship between the upper and lower airways and its contribution to T helper 2 (Th2)-skewed disease, such as AR and/or ECRS with asthma, has led us to this novel therapeutic strategy for a comprehensive approach to the treatment of upper airway inflammation with asthma.

## 1. Etiology of United Airway Disease

Eosinophilic airway inflammation such as allergic rhinitis (AR) or chronic rhinosinusitis with nasal polyposis (CRSwNP) is often associated with lower airway diseases, such as asthma. Particularly in the patient with aspirin-exacerbated respiratory disease, CRSwNP is accompanied by severe asthma [1]. The coexistence of both upper and lower airway disease is known as united airway disease, described as one airway, one disease. Diseases of the upper and lower airways share macroscopic pathologic characteristics as well as similar histological appearance in rhinitis and asthma [2]. In the Allergic Rhinitis and its Impact on Asthma (ARIA) guidelines, Bousquet et al. suggested that the upper and lower airways could be considered a single entity, supporting the united airways concept, but also highlighted some differences [3]. This concept involves a continuum of inflammation involving one common airway from the upper to the lower airway, and is considered as a heterogeneous disorder caused by allergic or nonallergic reproducible mechanisms and presents several phenotypes [4,5].

In recent decades, the prevalence of AR has markedly increased to almost 30% since the beginning of 2000 in the context of the Western lifestyle [6]. Recent studies have estimated that 30% of AR patients and 70% of asthma patients have comorbid asthma and AR, respectively [4,7]. Both types of inflammation, AR and asthma, develop in a unified morphological and functional unit and have similarities to allergen sensitization and the process of inflammation. In a cross-sectional multicenter study based on the Self-Assessment of Allergic Rhinitis and Asthma (SACRA) questionnaire (state of the impact of allergic rhinitis on asthma control, or SACRA study), Ohta et al. reported that asthma control was significantly impaired in AR patients and that significantly more AR patients had uncontrolled asthma than those without AR [7]. Previous surveys evaluating AR and asthma revealed that the long-term risk of developing asthma was three times higher in AR patients [8], and the incidence of asthma attacks was comparatively greater in asthma patients with AR [9]. In addition, Togias et al. reported an association between degree of severity of asthma and AR [10].

In CRSwNP, there is a marked infiltration of eosinophils into the nasal polyp. In Japan, CRSwNP with eosinophilia, referred to as eosinophilic rhinosinusitis (ECRS), is diagnosed using the Japanese Epidemiological Survey of Refractory Eosinophilic Chronic Rhinosinusitis (JESREC) scoring system [11]. ECRS is an intractable disease because of recurrences following multiple surgeries and is the major endotype of CRSwNP in the Western countries [12,13]. Its prevalence in Japan and Korea has increased in the last >20 years [14,15]. Likewise, ECRS, similar to AR, contributes to T helper 2 (Th2)-skewed pathology and is strongly associated with severe asthma [11,13]. Indeed, we have reported that fractionated exhaled nitric oxide reflective of disturbance of lung function was correlated with sinus computed tomography (CT) score based on the Lund–Mackay scale [16].

Recently, Giavina-Bianchi et al. suggested the consideration of united airway disease as airway hypersensitivity syndrome because rhinitis and asthma are chronic inflammatory diseases of the upper and lower airways and are induced and reproduced by allergic or nonallergic hypersensitivity reactions [5]. Moreover, Wu et al. described two inflammatory phenotypes, eosinophilic and non-eosinophilic, with a distinct clinical profile for nasal polyps and comorbid asthma, which is a common united airway disease [17]. This evidence indicates that phenotypes and endotypes in united airway disease must be classified by clinical features and molecular pathogenesis, respectively, in further studies.

Thus, the upper and lower airways must be integrated into the total airway, and a focus on the concept of united airway disease, with simultaneous treatment of both AR or ECRS and asthma, is required.

## 2. Relationships Among the Upper and Lower Airways and the Middle Ear in United Airway Disease

In united airway disease characterized by eosinophilic inflammation, there are close relationships between not only the upper and lower airway inflammation but also between the middle ear and airway disease [5,18,19]. However, there is less evidence regarding the interaction between otitis media and AR or ECRS. Nguyen et al. suggested that similar allergic inflammation linked AR and otitis media with effusion and that the middle ear is a part of the united airway in atopic individuals [18]. Seo et al. reported that eosinophilic otitis media was associated with asthma severity [20], and the treatment of asthma improved eosinophilic otitis media [19]. Although little is known about the precise mechanisms for these relationships, it is postulated that chemical mediators released during an allergic reaction in the nose affect the middle ear through the nasolacrimal duct and lacrimal system or indirectly via the blood stream [19,21]. 

**Figure 1:**
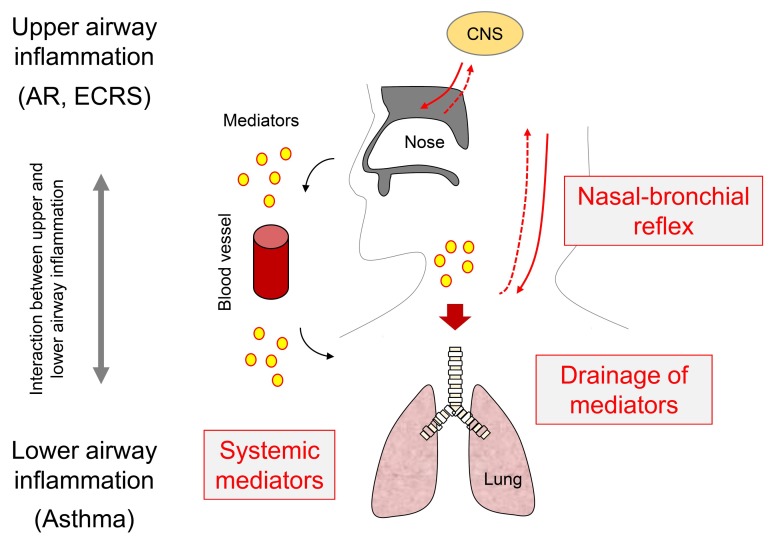
Schema of mechanisms of interaction between upper and lower airway inflammation. Red line and dot-line indicate parasympathetic and trigeminal nerve, respectively. AR, allergic rhinitis; CNS, central nervous system; ECRS, eosinophilic chronic rhinosinusitis.

There is more evidence regarding the relationship between asthma and AR or ECRS than for that between the ear and airway. In routine medical practice, otolaryngologists or pulmonologists often see improvements in lower airway inflammation following the treatment of upper airway inflammation, such as ECRS. Thus, this clinical experience indicates that there are interactions between the upper and lower airways, but this interaction cannot be simply explained by similarities to allergen sensitization and the process of inflammation in the total airway. Recent studies have described this interaction as follows: endoscopic sinus surgery (ESS) improved asthma symptoms as well as peak expiratory flow in patients with CRSwNP [22]; ESS improved scores in both the Mini Asthma Quality of Life Questionnaire (AQLQ) and the Asthma Control Test (ACT) using validated outcome metrics by 50% CRSwNP [23]; and ESS significantly improved the asthma symptoms, peak flow, and arbitrary medication scores, using the US Food and Drug Administration criteria [24]. Although these results suggest functional interactions between the upper and lower airways in united airway disease, little is known about the potential mechanisms for control. Currently, as shown in Figure 1, there are three possible mechanisms for the observed association between upper and lower airway inflammation, which are as follows: (1) decrease in postnasal drainage of inflammatory mediators from the upper to the lower airways; (2) reduction of systemic mediators disseminated by the upper airway; and (3) neural modulation via the nasal–bronchial reflex (NBR) [5].

## 3. Drainage and Systemic Propagation of Inflammatory Mediators from Nasal Inflammation

In a likely mechanism, drainage of the upper airway inflammatory mediators is a convincing mechanism, induced by the aspiration of inflammatory mediators in upper airway postnasal discharge into the lower airway [5,10,25]. In clinical practice, patients with respiratory symptoms such as coughing with disturbance of pulmonary function and/or increasing airway hyperresponsiveness (AHR) in the morning because of aspiration associated with overnight secretions resulting from upper airway disease are often encountered [3]. Moreover, Brugman et al. showed that the development of sinusitis in the sinusitis rabbit model was related to lower AHR, even after eliminating upper–lower airway communication [26]. In contrast, Bardin et al. used a human protocol of radioactivity in the lower airway 24 h following technetium 99-metastable (^99 m^Tc) injection into the maxillary sinuses of patients with CRS with asthma, reporting that seeding of the lower airways by mucopurulent secretions is unlikely to account for coexistent pulmonary disease [27]. Together, these studies suggest that this theory cannot sufficiently explain all mechanisms of this interaction.

Another theory describes the mechanism by which bone marrow-derived systemic inflammatory response and systemic mediators from upper airway inflammation are disseminated via the bloodstream. Interestingly, Braunstahl et al. reported that bronchial provocation in AR patients without asthma could induce allergic inflammation in the nose as well as increase the number of peripheral blood eosinophils [28]. They showed that nasal allergen provocation in AR patients could induce the expression of adhesion molecules and tissue eosinophilia in the upper and lower airways [29]. Finally, results of a study by Higashi et al. suggested that cysteinyl leukotriene overproduction might be involved in hyperplastic rhinosinusitis with nasal polyposis in asthma patients, noting significant decreases in the urinary leukotriene E4 concentrations after the sinus surgery in both aspirin-intolerant and aspirin-tolerant asthma patient [30].

## 4. Neural Modulation in United Airway Disease

**Figure 2:**
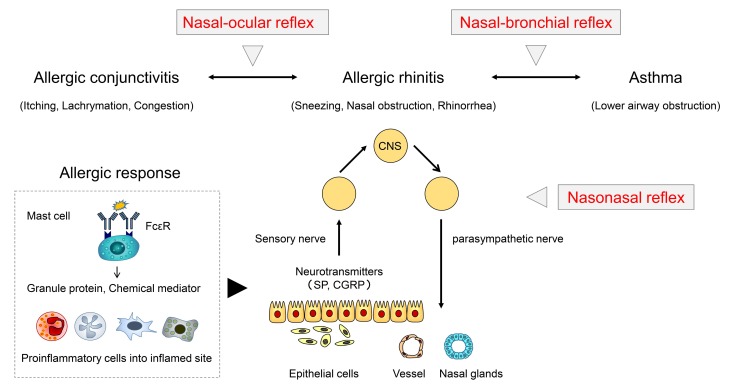
Neural pathway in allergic rhinitis; nasonasal reflex, nasal-ocular reflex, and nasal–bronchial reflex. CGRP, calcitonin gene-related peptide; SP, substance P; FcεR, Fc epsilon receptor

Nasal swelling of the mucosa and discharge through stimulation to the nasal vasculature and glands by inflammatory cascade in AR is regulated not only by direct effects of chemical mediators released from inflammatory cells and epithelial cells but also by neural modulation after specific antigen-antibody reaction [31,32]. In studies of AR patients who underwent Vidian neurectomy, Konno et al. defined the role of the neural network in AR [33,34]. In their investigations, signaling of the sensory nerve endings in the nose is transmitted to the central nervous system (CNS) via the trigeminal nerve; this signaling is then involved in the nasal vasculature and gland on the opposite side (the nasonasal reflex) through parasympathetic nerves (Figure 2).

Eye symptoms associated with AR were investigated by Baroody et al. [35,36]. In this mechanism, called the nasal-ocular reflex, a neurogenic network signals the CNS following trigeminal stimulation and partly contributes to the relationship between allergic conjunctivitis and AR (Figure 2) [35,36,37,38], whereas chemical mediators released during allergic reaction in the nose involved in allergic conjunctivitis may also contribute to this interaction via the bloodstream [38].

Notably, it is believed that this neurogenic network, called NBR, also exists between the upper and lower airways through the CNS by stimulation of the trigeminal in the nose and via an efferent pathway through a parasympathetic nerve such as the vagus nerve (Figure 1 and Figure 2) [5]. In humans, Corren et al. demonstrated that the nasal-allergic response by nasal provocation with allergen in AR patients enhanced AHR in the lower airway [39], and that nasal corticosteroid delivered into nasal cavity reversed this increased AHR in the lower airway associated with antigen exposure in AR and asthma patients [40]. Consistent with this, Bonay et al. reported that not only AHR but also the total number of eosinophils and eosinophil-cationic protein level in the sputum was increased after pollen challenge into the nose of patients with seasonal AR [41]. Furthermore, after ovalbumin sensitization mice, nasal ovalbumin (OVA) provocation using an aerosol rapidly induced AHR in the lung via the pulmonary upregulation of substance P (SP) and activation of neurokinin 1 receptors (NK-1R) [42]. However, whether signaling from the nose through a neural pathway between the upper and lower airways can promote chronic pulmonary inflammation and persistent AHR remains unclear. To clarify this, additional experiments are needed: For instance, investigations about relevance between neural inflammation by neurotransmitters, such as tachykinins (SP and neurokinins) [43,44] and NBR are required.

In conclusion, although all mechanisms for interaction between upper airway inflammation and asthma are difficult to explain using one theory, several possible mechanisms may overlap to produce this pathology.

## 5. A Novel Therapeutic approach to United Airway Disease

Recently, CRSwNP with a shift from predominantly neutrophilic toward eosinophilic airway inflammation has been dramatically increasing, especially in several Asian countries over the last 20 years [45]. Notably, prevalence of ECRS, considered a special and recalcitrant subtype of CRS, has been increased since more than 20 years [46]. Concurrently, treatment of asthma has switched from systemic to local corticosteroid administration such as inhaled corticosteroid (ICS) [47]. The possibility of this drastic therapeutic change might lead to an insufficient treatment of upper airway inflammation, while no epidemiological study about this relevance has been reported. If this is true, systemic corticosteroid administration for the simultaneous treatment of both asthma and AR or ECRS is a better approach to treating united airway disease. Nevertheless, ICS is still an attractive approach because it has fewer side effects than systemic corticosteroid administration, and ICS treatment dramatically decreases the risk of death from asthma [48]. Actually, in the meta-analysis, Lohia et al reported that intranasal corticosteroid medications improved pulmonary function, bronchial reactivity, asthma symptom scores, asthma-specific quality of life, and rescue medication use in patients with both AR and asthma [49], suggesting the efficacy of local corticosteroid therapy in united airway disease.

**Figure 3:**
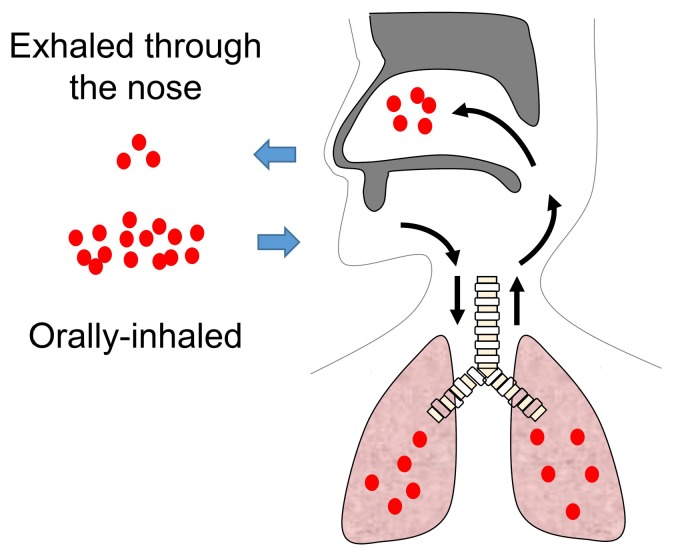
Schema of HFA-BDP ETN treatment; hydrofluoroalkane-134a-beclomethasone dipropionate (HFA-BDP) exhaled through the nose (ETN) treatment. Red dots indicate corticosteroids.

A novel approach focused on the concept of united airway disease is, therefore, needed for the simultaneous treatment of both upper and lower airway inflammation using ICS. To regulate united airway disease, as shown in Figure 3, we developed an original approach using the oral inhalation of fine particles of ICS, which are then exhaled through the nose (ETN) [50]. 

A benefit of ICS is that a sufficient number of fine particles are delivered in the nasal cavity as well as in the lower airway. In patients with uncontrolled, recurrent CRSwNP with asthma, including those treated with a combination of ICS to hydrofluoroalkane-134a-beclomethasone dipropionate via a metered-dose inhaler (HFA-BDP MDI) ETN treatment improved the sinus CT score defined by Lund–Mackay scale, airway obstruction evaluated by forced expiratory volume in 1 second (%FEV1) and forced expiratory flow between 25% and 75% of vital capacity (%FEF_25–75_) and the total number of peripheral eosinophils [50]. In addition, our blinded, placebo-controlled study revealed similar improvements with HFA-BDP MDI ETN treatment and placebo [51]. Interestingly, long-Acting Beta agonists (LABAs) such as formoterol and salmeterol have the effect of not only relaxing smooth muscle in the bronchi but also restoring corticosteroid sensitivity in patients with severe asthma [52], resulting in a synergistic effect. Currently, ICS/LABA therapy is often used in asthma patients. As expected, the ICS/LABA ETN treatment of patients with refractory ECRS with severe asthma results in improved nasal symptoms with asthma control [53]. Thus, we suggest that simultaneous treatment in upper and lower airway by HFA-BDP MDI ETN enhances therapeutic effect by blocking interaction between upper and lower airway inflammation.

Recently, another inhaled bronchodilator using an anticholinergic drug that blocks acetylcholine (ACh) receptors, such as a long-acting muscarinic antagonist (LAMA), was used to treat chronic obstructive pulmonary disease as well as asthma, and several studies reported that, in asthma patients, ICS combined with LAMA (ICS/LAMA) has greater efficacy and safety than the same dose of ICS alone [54,55]. However, because LAMA exerts its bronchodilator effect via the inhibition of muscarinic but not nicotinic receptors [56], the use of an anticholinergic compound must be carefully evaluated in united airway disease. Therefore, the effect of ICS/LAMA in united airway disease can be clarified by close patient follow-up, and it also has the potential for improving asthma.

An interesting future therapeutic strategy in united airway disease focuses on regulation by neural inflammation via NBR in an interaction between upper and lower airway disease. Rosas-Ballina et al. recently reported that the signaling of ACh receptors from the parasympathetic nerves modulates the immune reaction [57]. Thus, future investigations may elucidate the mechanism for the signaling of ACh receptors, including both muscarinic and nicotinic receptors, by ACh released from parasympathetic nerves.

## 6. Conclusions

We have reviewed the etiology of united airway disease, focusing on the interaction between upper and lower airway inflammation and, in particular, the mechanism by which neural pathways are regulated. We suggest that next-generation therapy for united airway disease should involve the regulation of neural inflammation and that this approach will clinical impact.
